# A joint analysis of metabolomic profiles associated with muscle mass and strength in Caucasian women

**DOI:** 10.18632/aging.101574

**Published:** 2018-10-14

**Authors:** Qi Zhao, Hui Shen, Kuan-Jui Su, Qing Tian, Lan-Juan Zhao, Chuan Qiu, Timothy J. Garrett, Jiawang Liu, David Kakhniashvili, Hong-Wen Deng

**Affiliations:** 1 Department of Preventive Medicine, College of Medicine, University of Tennessee Health Science Center, Memphis, TN 38163, USA; 2 Tulane Center of Bioinformatics and Genomics, Department of Global Biostatistics and Data Science, Tulane University School of Public Health and Tropical Medicine, New Orleans, LA 70112, USA; 3 Southeast Center for Integrated Metabolomics Core, University of Florida, Gainesville, FL 32610, USA; 4 Medicinal Chemistry Core, Office of Research, University of Tennessee Health Science Center, Memphis, TN 38163, USA; 5 Department of Pharmaceutical Sciences, College of Pharmacy, University of Tennessee Health Science Center, Memphis, TN 38163, USA; 6 Proteomics and Metabolomics Core, Office of Research, University of Tennessee Health Science Center, Memphis, TN 38163, USA; 7 School of Basic Medical Science, Central South University, Changsha, Hunan 410013, China; * Equal contribution

**Keywords:** lean mass, grip strength, metabolite, metabolomics, muscle, sarcopenia

## Abstract

Both loss of muscle mass and strength are important sarcopenia-related traits. In this study, we investigated both specific and shared serum metabolites associated with these two traits in 136 Caucasian women using a liquid chromatography-mass spectrometry method. A joint analysis of multivariate traits was used to examine the associations of individual metabolites with muscle mass measured by the body mass index-adjusted appendicular lean mass (ALM/BMI) and muscle strength measured by hand grip strength (HGS). After adjusting for multiple testing, nine metabolites including two amino acids (aspartic acid and glutamic acid) and an amino acid derive (pipecolic acid), one peptide (phenylalanyl-threonine), one carbohydrate (methyl beta-D-glucopyranoside), and four lipids (12S-HETRE, arachidonic acid, 12S-HETE, and glycerophosphocholine) were significant in the joint analysis. Of them, the two amino acids (aspartic acid and glutamic acid) and two lipids (12S-HETRE and 12S-HETE) were associated with both ALM/BMI and HGS, and the other five were only associated with ALM/BMI. The pathway analysis showed the amino acid metabolism pathways (aspartic acid and glutamic acid) might play important roles in the regulation of muscle mass and strength. In conclusion, our study identified novel metabolites associated with sarcopenia-related traits, suggesting novel metabolic pathways for muscle regulation.

## Introduction

Age-related sarcopenia has become a major contributor to the risk of physical frailty, functional impairment, poor health-related quality of life, and premature death among the elderly [1–3]. It has emerged as a major health concern and resulted in a huge economic burden as the older population rapidly grows in the US [1]. In 2000, the direct medical costs attributable to sarcopenia was estimated to be about $18.5 billion in the US alone [1]. However, the etiology of sarcopenia remains largely unknown, and there are no approved medications for treating this disorder. Therefore, there is an urgent need for understanding the mechanisms of sarcopenia with the aim of improving its prevention, diagnosis, treatment, and prognosis. Although sarcopenia was originally considered to be solely loss of muscle mass, recently developed definitions of sarcopenia have incorporated both loss of muscle mass and muscle function [4–8]. It is supported by the facts that age-related decline in muscle strength does not parallel with the decline in muscle mass [9] and muscle weakness is a more consistent predictor for impaired physical performance, functional limitation, and physical disability compared to the loss in muscle mass [10]. This consensus highlights the necessity and importance of considering different physiological components of sarcopenia for obtaining a fundamental understanding of the complex biological mechanism underlying the decline in muscle mass and function.

Metabolomics is an emerging approach to systematically profile small molecules in biofluids, cells, and tissues [11]. Because metabolites represent the downstream expression of genome, transcriptome, and proteome, their study is hence most powerful to reveal inherent omics variation closest to the disease risk/phenotype [11]. Some metabolomics studies of muscle mass have been reported, recently [12–17]. Some amino acids (e.g., leucine, isoleucine, and glutamic acid) [12,16], lipids [14], and metabolites related to gut bacterial metabolism have been linked with muscle mass. However, very few metabolomic studies of muscle function have been conducted [13], and no studies have considered both muscle mass and muscle function at the same time. In fact, loss of muscle mass and strength starts at approximately 30 years of age. However, most of the previous metabolomic studies were conducted in the older population aged 60 years or older, which might fail to provide early metabolic markers for the loss of muscle mass and function.

The overall goal of this exploratory study was to identify novel metabolites and metabolic pathways associated with muscle mass and strength, providing metabolic markers for the early prediction of sarcopenia. We used an untargeted metabolomics approach and jointly analyzed the muscle mass and muscle strength to identify specific and shared metabolic factors for these two key sarcopenia traits in a sample of Caucasian women of a relatively young age.

## RESULTS

Characteristics of the sample of 136 women are shown in Table 1. The study participants were relatively young with an average age (SD) of 31.5 (5.1) years. The mean BMI, 26.0 kg/m^2^, was overweight. Over 35% of the participants were current smokers. On average, the participants had physical exercise about three times a week.

**Table 1:** Characteristics of the study subjects (N=136).

**Variable**	**Mean (SD) or Percentage**
Age, year	31.5 (5.1)
Weight, kg	70.3 (21.4)
Height, cm	164.6 (6.4)
BMI, kg/m^2^	26.0 (7.5)
Current smoking, %	35.3
Alcohol drinking, gram/day	36.5 (52.4)
Physical activity, times/week	3.1 (2.2)
Dairy intake, cups/day	1.6 (1.3)
ALM/BMI index	0.78 (0.12)
Hand grip strength, kg	27.0 (8.4)

ALM, appendicular lean mass; BMI, body mass index; SD, standard deviation.

**Figure 1:**
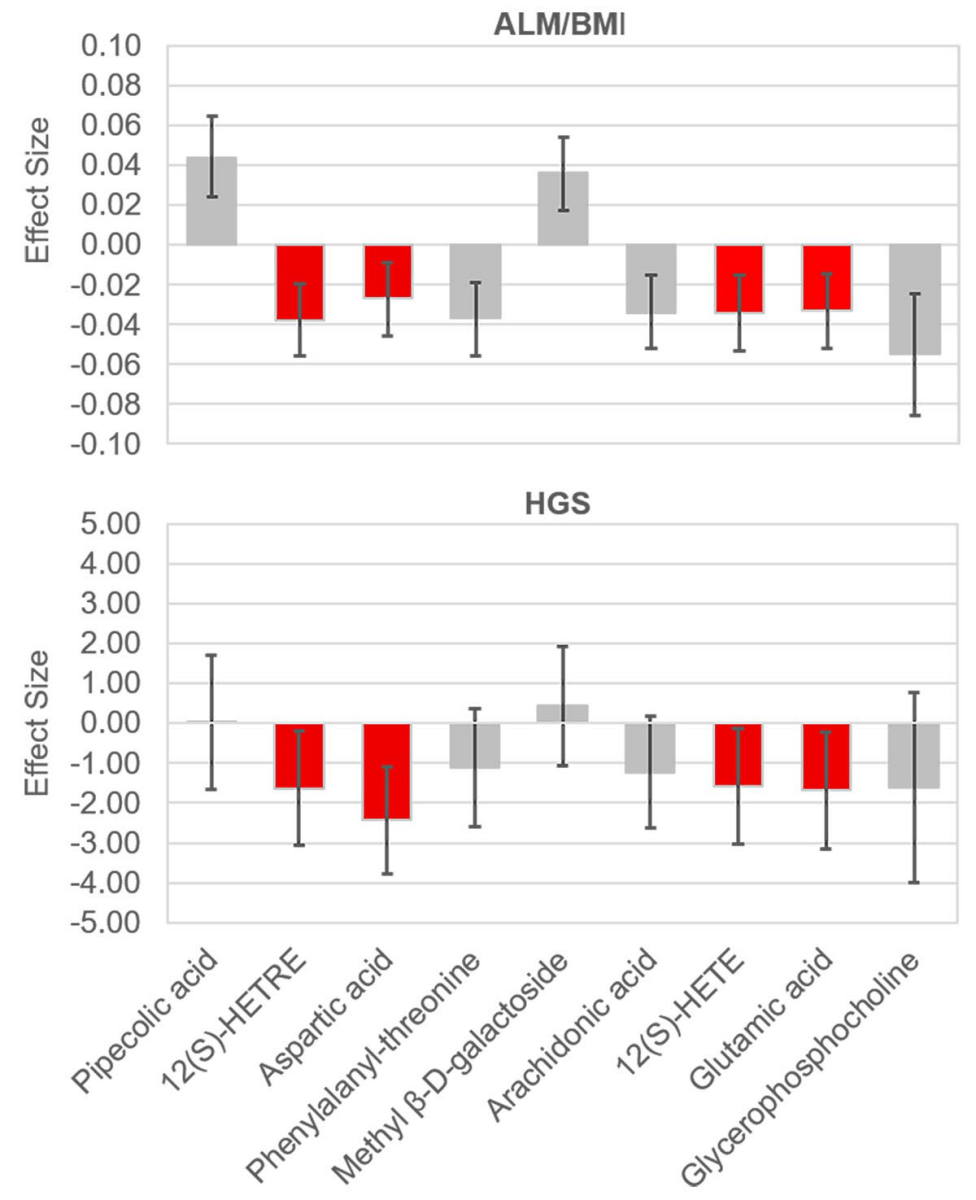
**Effect sizes of the significant metabolites on ALM/BMI and HGS.** The effects were associated with one standard deviation increase in the relative abundance of a metabolite. The bars show the 95% confidence internal for the effect of each metabolite. The metabolites which were significantly associated with both traits are lighted in red. ALM, appendicular lean mass; BMI, body mass index; HGS, hand grip strength.

**Figure 2:**
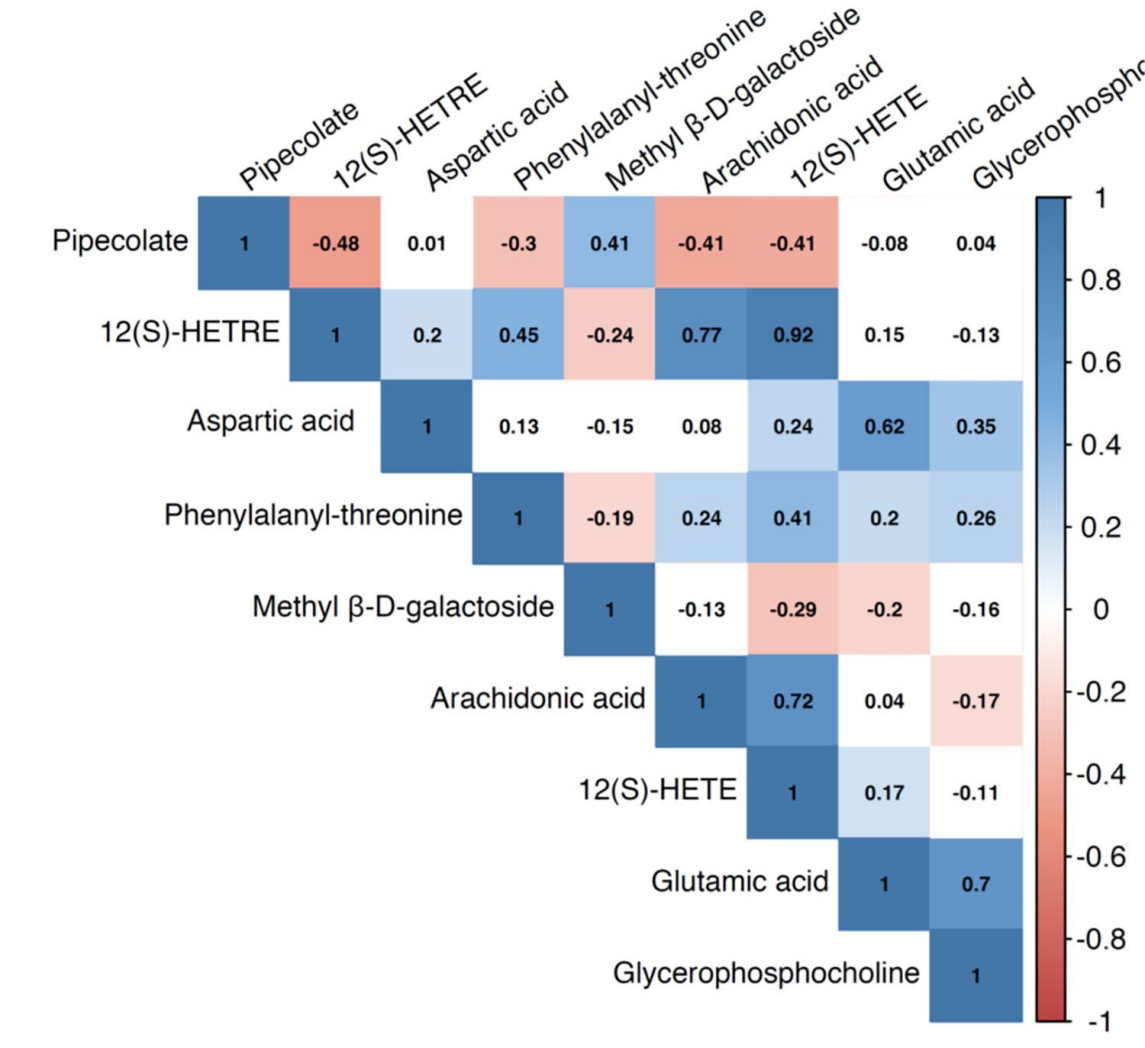
Pairwise correlation coefficients among the metabolites significantly associated with sarcopenia traits.

We identified a total of 206 metabolites with known identities and passed quality control using the LC-MS metabolomics platform. In the joint analysis, nine metabolites were significantly associated with muscle mass and muscle strength after adjusting for multiple testing (FDR q < 0.05) (Table 2). They belong to the chemical classes of organic acids, amino acids, peptides, carbohydrates, and lipids, respectively. All the nine metabolites were significantly associated with ALM/BMI. Among them, two amino acids (aspartate and glutamic acid) and two lipids [12(S)-HETRE and 12(S)-HETE] were also associated with HGS. The directions of the associations of these four metabolites with ALM/BMI and HGS were consistent (Figure 1). Another two lipids, arachidonic acid and glycerophosphocholine, were negatively associated with ALM/BMI as well. In contrast, the carbohydrate, methyl β-D-galactoside, was positively associated with ALM/BMI. Figure 2 shows the correlations among the significant metabolites. Three metabolites, 12(S)-HETRE, 12(S)-HETE, and arachidonic acid, were highly correlated each other with the correlation coefficients over 0.7. We didn’t observe any significant pairwise interactions among these significant metabolites in the relation to ALM/BMI or HGS after adjusting for multiple testing (Supplementary Table 1).

**Table 2:** The metabolites significantly associated with ALM/BMI or HGS.

**Metabolite**	**Class**	**m/z**	**RT**	***P* value**		
				**Joint analysis**	**ALM/BMI**	**HGS**
Pipecolic acid	Organic acid	130.0862	1.25	7.69×10^-5^	4.32×10^-5^	9.86×10^-1^
12(S)-HETRE	Lipid	321.2438	14.17	9.34×10^-5^	5.18×10^-5^	2.56×10^-2^
Aspartic acid	Amino Acid	134.0446	0.73	2.25×10^-4^	5.33×10^-3^	4.29×10^-4^
Phenylalanyl-threonine	Peptide	267.1334	6.43	4.85×10^-4^	1.43×10^-4^	1.35×10^-1^
Methyl β-D-galactoside	Carbohydrate	217.0679	1.02	5.32×10^-4^	1.60×10^-4^	5.67×10^-1^
Arachidonic acid	Lipid	305.2467	14.8	7.86×10^-4^	2.87×10^-4^	8.30×10^-2^
12(S)-HETE	Lipid	319.2275	13.8	1.00×10^-3^	6.23×10^-4^	3.11×10^-2^
Glutamic acid	Amino Acid	146.0458	0.76	1.02×10^-3^	7.63×10^-4^	2.32×10^-2^
Glycerophosphocholine	Lipid	258.1094	0.76	1.71×10^-3^	4.91×10^-4^	1.81×10^-1^

ALM, appendicular lean mass; BMI, body mass index; HGS, hand grip strength; RT, retention time.

In the pathway analysis, eight metabolic pathways were significant in the enrichment analysis (*P* values < 0.05) (Table 3). Of them, seven pathways include glutamic acid and/or aspartic acid. The impact values of the two amino acids on these pathways ranged widely (from almost 0 to 0.44). In addition, we found only pipecolic acid and glutamic acid significantly associated with age after adjusting for multiple covariables (*P* values < 0.05) (Table 4).

**Table 3:** Pathway analysis results of trait-related metabolites.

**Pathways**	**Matched Metabolites**	***P* value ^a^**	**Impact value ^b^**
Alanine, aspartate and glutamate metabolism	Glutamic acid; Aspartic acid	9.36×10^-4^	4.4×10^-1^
Nitrogen metabolism	Glutamic acid; Aspartic acid	2.48×10^-3^	6.7×10^-4^
Histidine metabolism	Glutamic acid; Aspartic acid	3.15×10^-3^	5.1×10^-4^
Aminoacyl-tRNA biosynthesis	Glutamic acid; Aspartic acid	9.01×10^-3^	1.1×10^-2^
Arginine and proline metabolism	Glutamic acid; Aspartic acid	9.49×10^-3^	3.6×10^-2^
D-Glutamine and D-glutamate metabolism	Glutamic acid	2.27×10^-2^	1.1×10^-1^
Cyanoamino acid metabolism	Aspartic acid	3.28×10^-2^	0
Ether lipid metabolism	Glycerophosphocholine	4.69×10^-2^	0

^a.^ *P* values from the pathway enrichment analysis.

^b.^ Impact values from the pathway topology analysis.

**Table 4:** Correlations between trait-related metabolites and age.

**Metabolite**	**Correlation coefficient**	***P* value**	**Partial correlation coefficient ^a^**	***P* value**
Pipecolic acid	-0.28	1.24×10^-3^	-0.21	1.77×10^-2^
12(S)-HETRE	0.13	1.23×10^-1^	0.09	3.33×10^-1^
Aspartic acid	-0.07	4.55×10^-1^	-0.08	3.50×10^-1^
Phenylalanyl-threonine	0.08	3.84×10^-1^	0.01	8.67×10^-1^
Methyl β-D-galactoside	-0.19	3.21×10^-2^	-0.13	1.57×10^-1^
Arachidonic acid	0.11	2.17×10^-1^	0.08	3.43×10^-1^
12(S)-HETE	0.14	1.10×10^-1^	0.10	2.38×10^-1^
Glutamic acid	-0.11	2.07×10^-1^	-0.17	4.98×10^-2^
Glycerophosphocholine	-0.10	2.34×10^-1^	-0.17	5.23×10^-2^

^a.^ The covariables including body mass index, smoking, alcohol drinking, physical activities, and diary intakes were adjusted.

## DISCUSSION

The present study, for the first time, jointly investigated the metabolites significantly associated with two important sarcopenia traits, muscle mass and strength. We identified nine metabolites, mainly amino acids and lipids, associated with muscle mass, four of which were also associated with muscle strength. Most of the identified metabolites represented novel findings compared to previous reports, highlighting the importance of amino acids and lipids in the regulation of muscle mass and function.

Previous studies have shown that amino acids are potent stimulators of muscle protein synthesis in both the young and elderly [18–20]. Circulating (plasma or serum) amino acids, especially branched chain amino acids (such as leucine, isoleucine, and valine), have been associated with muscle mass in previous studies [12,17]. We identified two non-branched chain amino acids, aspartic acid and glutamic acid, were significantly associated with both muscle mass and muscle strength. The finding is consistent with prior evidence regarding the association of glutamic acid with lean mass identified in a sample of UK women [16]. However, this is the first time to report that glutamic acid was also associated with muscle strength in human studies. Glutamate (the anion of glutamic acid) in skeletal muscle participates in various metabolic pathways, such as glutathione synthesis, insulin production, tricarboxylic acid cycle, and purine nucleotide cycle. Muscle glutamate metabolism is disturbed in many conditions, such as hypoxia and oxidative stress [21]. Aspartate (the anion of aspartic acid) has been shown to inhibit inflammation-induced muscle loss through regulating phosphorylation of Akt, AMPKα, and FOXO1 in a piglet model [22]. Our pathway analysis further highlights potential important roles of several amino acid metabolic pathways involving both glutamic acid and aspartic acid, such as the pathways of alanine, aspartate, and glutamate metabolism as well as arginine and proline metabolism, in muscle regulation.

Pipecolic acid, the most significant metabolite associated with muscle mass in this study, is also an amino acid-related molecule. It is a metabolite of lysine which is an essential amino acid and very abundant in muscle tissue. It has been recently reported that oral administration of lysine could suppress proteolysis in skeletal muscles of fasted rats [23] and continuous feeding of a lysine-rich diet might increase muscle mass [24]. Supplementation of lysine in rats could reduce the upregulated autophagy activity of skeletal muscle caused by a low-protein diet and activate the Akt/mTOR signaling pathways [24]. As a metabolite of lysine, pipecolic acid also could stimulate the rates of protein synthesis, contributing to the effect of lysine on protein turn over in skeletal muscle [25]. We found that serum pipecolic acid was positively associated with muscle mass and muscle strength, but negatively associated with age. These findings may suggest pipecolic acid is a potential early marker for sarcopenia.

There is increasing evidence that supports a role of lipids and their derives in the regulation of skeletal muscle mass and function through their ability to modulate muscle cell growth, proliferation, and/or differentiation [26]. In particular, a recent study has observed significant changes in lipid mediators, a class of bioactive metabolites of the essential polyunsaturated fatty acids (PUFA), in skeletal muscles with aging in mice [27]. We identified one fatty acid (arachidonic acid) and two fatty acid derivatives [12(S)-HETE and 12(S)-HETRE] that were associated with both muscle mass and strength. Arachidonic acid, a PUFA, is the precursor of prostaglandins, leukotrienes, and related compounds, which have important roles in inflammation and in the regulation of immunity [28]. 12(S)-HETE and 12(S)-HETRE are derivatives of arachidonic acid and dihomo-λ-linolenic acid metabolism through the 12-lipoxygenase pathway, respectively [29]. We found the levels of the three molecules were highly correlated with each other in the serum and negatively associated with muscle mass and strength, suggesting they might be risk factors for the loss of muscle mass and function. However, previous studies showed that arachidonic acid could enhance *in vitro* skeletal muscle cell growth via a COX-2-dependent pathway [30]. Also, arachidonic acid supplementation could augment strength-training-induced adaptations in resistance-trained males [31]. These inconsistent findings might indicate the complexity of the role of arachidonic acid in skeletal muscle regulation. Their effects might vary by sex, age, and different pathological conditions. Therefore, it will be worth conducting metabolomics studies which target the whole pathway of arachidonic acid metabolism and even related pathways of fatty acid metabolism to investigate their comprehensive role in regulating muscle mass and function.

Arachidonic acid and its lipid mediators, including prostaglandin F_2α_ (PGF_2α_), PGE_2_, and PGA_2_, decrease with aging in mice gastrocnemius muscles [27]. However, just like most of other trait-related metabolites identified in this study, we did not observe significant correlation between serum arachidonic acid and age. This might be because of differential changing of these metabolites in the muscle tissue and blood with aging. The relatively limited age range of the study population (from 20 to 40 years) might also undermine the study power to detect the change in the metabolites with aging. Longitudinal studies are necessary to clarify the changes in these metabolites with aging in the future. Furthermore, we identified another lipid, glycerophosphocholine, might be a risk factor for the loss of muscle mass. Very few studies have investigated the role of glycerophosphocholine in muscle regulation. Only a rat study showed an increased level of glycerophosphocholine in aged gastrocnemius [32].

We identified methyl β-D-galactoside, a member of carbohydrates, was positively associated with muscle mass. Methyl β-D-galactoside has been found in cereals and cereal products. Previous studies showed that carbohydrate consumption could stimulate muscle protein synthesis after resistance exercise [33]. On the contrary, other studies augured that the muscle protein increase during carbohydrate supplementation alone following resistance exercise is mainly through reduction at protein degradation rates rather than through increasing protein synthesis. Little is known about the mechanisms through which methyl β-D-galactoside regulates muscle mass. Further investigations are needed to clarify its role in muscle mass regulation.

Our study has several strengths. First, we considered both muscle mass and muscle strength which reflect important pathophysiological aspects of sarcopenia, in examining the associations with blood metabolites. Second, we used DXA to measure body compositions, which is widely used and well accepted to assess muscle mass [34]. Third, the non-hypothesis-driven untargeted metabolomics approach enabled the study to discover novel metabolites and pathways involved in muscle regulation. Fourth, adjustment for potential confounding factors and multiple testing were conducted to minimize false-positive findings. Finally, we identified both specific and shared metabolites for muscle mass and muscle strengths, providing important knowledge for better understanding the pathogenesis of sarcopenia. However, some limitations should be considered in interpreting the study findings. Specifically, the causal relationship between identified metabolites and sarcopenia traits could not be inferred because of the cross-sectional study design. Also, the relatively small sample size may limit the statistical power to identify sarcopenia-trait related metabolites with small effects. However, this pilot study was developed to generate hypotheses for specific/shared metabolites for muscle mass and strength. Further studies are needed to confirm our findings and clarify the potential molecular mechanisms of the blood metabolites associated with sarcopenia traits.

In summary, we identified trait-specific or shared metabolites associated with muscle mass and strength. Our results highlight the importance of multiple metabolic pathways of amino acids and lipids in the regulation of muscle mass and function. Future studies targeting these pathways may lead to a better understanding of the pathogenesis of sarcopenia and identification of biomarkers for early prediction and prevention of this disorder.

## MATERIALS AND METHODS

### Study subjects

A total of 136 Caucasian women participants were from the ongoing Louisiana Osteoporosis Study (LOS) (from 2011 to current), which aims to build a large sample pool and database for investigating genetic and environmental factors for osteoporosis in Southern Louisiana. The inclusion and exclusion criteria of LOS have been described in our previous study [35]. All the study subjects included in this study were aged between 20 and 40 years old. Individuals who were pregnant, had a bilateral oophorectomy, or had any chronic conditions (such as diabetes mellitus, renal failure, liver failure, lung disease, gastrointestinal disease, and inherited bone disease) were excluded from the current study. The study was approved by the institutional review board, and a written consent form was signed by each participant before any data and bio-sample collection.

### Clinical measurements

All participants completed an interviewer-assisted comprehensive questionnaire to collect demographic information, lifestyle (including smoking, drinking, and physical activity), dietary factors (including dairy consumption), reproductive and medical history [35]. Weight was measured in light indoor clothing using a calibrated balance beam scale, and height was measured using a calibrated stadiometer without shoes. Body mass index (BMI) was calculated as weight (kg) divided by height squared (m^2^).

Total body and regional measures of lean mass, fat mass, and bone mineral content were acquired using a dual-energy X-ray absorptiometry (DXA) machine (Hologic Inc., Bedford, MA) by trained and certified research staff. The machine was calibrated daily, and software and hardware were kept up-to-date during the data collection process. More details on data quality control including the usual covariation for repeated measures have been reported previously [35]. Appendicular lean mass (ALM) was calculated as the sum of lean mass in the arms and legs. The BMI-adjusted ALM (ALM/BMI) was used to assess individuals’ muscle mass in the study [8]. Hand grip strength (HGS) was measured using the Jamar 1 hand-held dynamometer (TEC Inc., Clifton, NJ). Two measurements of maximum strength were taken at both hands. The maximum grip strength value was used to assess an individual’s muscle strength.

### Metabolomic analyses

The liquid chromatography-mass spectrometry (LC-MS) based metabolomics platform developed by Dr. Garrett’s lab in the Southeast Center for Integrated Metabolomics at University of Florida was used to perform the metabolomic analyses. The laboratory protocols have been previously described [36]. Briefly, frozen serum samples (-80 °C) were thawed at room temperature. Each serum sample (100 μL) was mixed with 20 μL internal standard mix followed by vortex mixing for 20 s. Next, 800 μL of acetonitrile: acetone:methanol (8:1:1, v: v: v) was added and centrifuged at 20,000×g for 10 min at <10°C to remove proteins. The supernatant (250 μL) was transferred to a new 1 mL Eppendorf tube and dried under a gentle stream of nitrogen (Organomation Associates, Berlin, MA, USA). The dried sample was reconstituted in 100 μL of 0.1% formic acid in water containing 4 injection standards (T-Boc amino acids) and placed in an ice bath for 10-15 min followed by centrifugation at 20,000×g for 5 min at <10°C to remove any debris.

A Thermo Q-Exactive High-Resolution Mass Spectrometer (Thermo Fisher Scientific, Fremont, CA) coupled with a Dionex UHPLC (Dionex Corporation, Sunnyvale, CA) was used to conduct the metabolomic analysis. All the samples were analyzed in both positive and negative ion modes with heated electrospray ionization. The mass resolution was 35,000 at m/z 200 with a mass accuracy of less than 5 ppm in positive mode and less than 10 ppm in negative mode. Separation was achieved on an ACE C18-PFP column (100x2.1mm, 2µm) with 0.1% formic acid in water as mobile phase A and acetonitrile as mobile phase B with a column temperature of 25 °C. The flow rate was 350 µL/min with a total run time of 20 min.

Alignment and feature finding was performed using the open source software MZmine [37]. Metabolite identification was performed by searching an internal retention time library of over 600 compounds. Peaks in the MS were quantified using integrated peak height. Raw area counts for each metabolite in every sample was first normalized to the sum of all injection internal standards to correct for subtle injection differences, and then normalized to the total signal of each sample. The batch correction was performed using well-established Bayes method for microarray data by continually running the neat and pooled quality control (QC) samples. Metabolites with missing rates > 20% or coefficients of variation > 20% were excluded from further analyses. Imputation of missing data was performed using the R package ‘missForest’ [38]. The relative abundance data of metabolites were further log transformed and autoscaled to have zero mean and unit variance (z scores) using the R package ‘specmine’.

### Statistical analyses

To increase the study power and decrease the multiple testing burden [39], we conducted a joint analysis of multivariate phenotypes to examine the associations of metabolites with sarcopenia traits (ALM/BMI and HGS). The null hypothesis (H_0_) of the joint analysis was that none of the traits was associated with the tested metabolite. At least one trait associated with the metabolite would reject the null hypothesis. The R package ‘systemfit’ was used to conduct the joint analysis [40]. The seemingly unrelated regression of ‘systemfit’ was conducted to estimate the coefficients of ALM/BMI and HGS with the metabolite, respectively, and the F-statistics were used to test the null hypothesis. Potential confounding factors, including age, BMI (for HGS only), smoking, alcohol drinking, physical activities, and diary intakes, were adjusted when examining the associations of ALM/BMI and HGS with metabolites. Considering the substantial correlations among metabolites, we used the false discovery rate (FDR) method to adjust for multiple testing. A metabolite with an FDR q-value < 0.05 was considered significant [41]. To evaluate the independence of significant metabolic associations, we estimated the pairwise Pearson correlations between all metabolites significantly associated with these traits. In addition, we examined the pairwise interaction effects of significant metabolites on ALM/BMI and HGS. To identify potential pathways represented by the significant metabolites, we conducted a pathway analysis which integrated enrichment analysis and pathway topology analysis using the web tool MetaboAnalyst [42]. The topology analysis takes into account the position of a metabolite in the pathway structure and provides an impact value, ranging from 0 (minimum impact) to 1 (maximum impact), for each tested metabolite. The correlation coefficients between significant metabolites and age without or with adjusting for covariables (including BMI, smoking, alcohol drinking, physical activities, and diary intakes) were also calculated to examine whether these metabolites changed with aging.

## SUPPLEMENTARY MATERIAL

Supplementary Table 1
