# Supplementary material for: A joint analysis of metabolomic profiles associated with muscle mass and strength in Caucasian women
Source: Aging (Albany NY). 2018 Oct 14;10(10):2624–35. doi: 10.18632/aging.101574 (PMC6224264; doi:10.18632/aging.101574)
Supplement: Supplementary Table 1 [file aging-10-101574-s001.docx]

**Supplementary Table 1. The significance for pairwise interaction effects.**

| **Metabolite A** | **Metabolite B** | ***P* value for ALM/BMI** | ***P* value for HGS** |
| --- | --- | --- | --- |
| Phenylalanyl-threonine | Pipecolic acid | 4.64E-01 | 7.30E-01 |
| Phenylalanyl-threonine | Glycerophosphocholine | 6.33E-02 | 6.96E-01 |
| Phenylalanyl-threonine | Aspartic acid | 2.50E-01 | 6.91E-01 |
| Phenylalanyl-threonine | Methyl β-D-galactoside | 2.76E-01 | 6.38E-01 |
| Phenylalanyl-threonine | Arachidonic acid | 4.52E-01 | 1.69E-01 |
| Phenylalanyl-threonine | Glutamic acid | 7.15E-01 | 4.45E-01 |
| Phenylalanyl-threonine | 12(S)-HETE | 2.17E-01 | 3.30E-01 |
| Phenylalanyl-threonine | 12(S)-HETRE | 7.44E-01 | 2.21E-01 |
| Pipecolic acid | Glycerophosphocholine | 6.60E-01 | **1.74E-02** |
| Pipecolic acid | Aspartic acid | 1.35E-01 | 1.74E-01 |
| Pipecolic acid | Methyl β-D-galactoside | 8.50E-01 | 9.88E-01 |
| Pipecolic acid | Arachidonic acid | 7.35E-01 | 8.34E-01 |
| Pipecolic acid | Glutamic acid | 1.42E-01 | 2.27E-01 |
| Pipecolic acid | 12(S)-HETE | 6.69E-01 | 4.10E-01 |
| Pipecolic acid | 12(S)-HETRE | 8.98E-01 | 6.81E-01 |
| Glycerophosphocholine | Aspartic acid | 9.25E-02 | 9.57E-01 |
| Glycerophosphocholine | Methyl β-D-galactoside | 5.47E-01 | **4.14E-03** |
| Glycerophosphocholine | Arachidonic acid | 6.87E-01 | 8.46E-01 |
| Glycerophosphocholine | Glutamic acid | 1.69E-01 | 1.18E-01 |
| Glycerophosphocholine | 12(S)-HETE | 8.23E-01 | 2.06E-01 |
| Glycerophosphocholine | 12(S)-HETRE | 7.30E-01 | 2.73E-01 |
| Aspartic acid | Methyl β-D-galactoside | 2.64E-01 | 2.90E-01 |
| Aspartic acid | Arachidonic acid | 1.34E-01 | 8.02E-01 |
| Aspartic acid | Glutamic acid | 5.73E-01 | 8.40E-01 |
| Aspartic acid | 12(S)-HETE | 2.59E-01 | 2.10E-01 |
| Aspartic acid | 12(S)-HETRE | 1.32E-01 | 1.09E-01 |
| Methyl β-D-galactoside | Arachidonic acid | 6.13E-01 | **2.57E-02** |
| Methyl β-D-galactoside | Glutamic acid | 2.51E-01 | **1.46E-02** |
| Methyl β-D-galactoside | 12(S)-HETE | 5.97E-01 | 2.34E-01 |
| Methyl β-D-galactoside | 12(S)-HETRE | 9.28E-01 | 2.40E-01 |
| Arachidonic acid | Glutamic acid | 1.99E-01 | 5.88E-01 |
| Arachidonic acid | 12(S)-HETE | 2.29E-01 | 7.19E-01 |
| Arachidonic acid | 12(S)-HETRE | 2.22E-01 | 2.58E-01 |
| Glutamic acid | 12(S)-HETE | 6.44E-01 | 2.62E-01 |
| Glutamic acid | 12(S)-HETRE | 3.13E-01 | 4.09E-01 |
| 12(S)-HETE | 12(S)-HETRE | 3.04E-01 | 3.91E-01 |
